# A multi-task learning approach combining regression and classification tasks for joint feature selection

**DOI:** 10.1038/s41598-026-43551-3

**Published:** 2026-04-17

**Authors:** Han Cao, Sivanesan Rajan, Bianka Hahn, Ersoy Kocak, Manuel Brenner, Florian Hess, Roman Schefzik, Daniel Durstewitz, Georgia Koppe, Emanuel Schwarz, Verena Schneider-Lindner

**Affiliations:** 1 Department of Theoretical Neuroscience, Medical Faculty, Central Institute of Mental Health, Heidelberg University, Mannheim, Germany; 2 Department of Psychiatry and Psychotherapy, Medical Faculty, Central Institute of Mental Health, Heidelberg University, Mannheim, Germany; 3 Hector Institute for Artificial Intelligence in Psychiatry, Medical Faculty Mannheim, Central Institute of Mental Health, Heidelberg University, Mannheim, Germany; 4 Department of Anesthesiology, Surgical Intensive Care Medicine and Pain Medicine, Medical Faculty Mannheim, Heidelberg University, Theodor-Kutzer-Ufer 1-3, 68167 Mannheim, Germany; 5 Interdisciplinary Center for Scientific Computing, Heidelberg University, Mathematikon Im Neuenheimer Feld 205, 69120 Heidelberg, Germany; 6 Faculty of Physics and Astronomy, Heidelberg University, Im Neuenheimer Feld 226, 69120 Heidelberg, Germany

**Keywords:** Machine learning, Biomarker identification, Sepsis, Molecular psychiatry, Schizophrenia, Data integration, Data mining, Machine learning

## Abstract

Multi-task learning (MTL) is a learning paradigm that enables the simultaneous training of multiple communicating algorithms, and has been widely applied in the biomedical analysis for shared biomarker identification. Although MTL has successfully supported either regression or classification tasks, incorporating mixed types of tasks into a unified MTL framework remains challenging, especially in biomedicine, where it can lead to biased biomarker identification. To address this issue, we propose an improved method of multi-task learning, MTLComb, which balances the weights of regression and classification tasks to promote unbiased biomarker identification. We demonstrate the algorithmic efficiency and clinical utility of MTLComb through analyses on both simulated data and actual biomedical studies pertaining to sepsis and schizophrenia. The code is available at https://github.com/transbioZI/MTLComb.

**Supplementary Information:**

The online version contains supplementary material available at 10.1038/s41598-026-43551-3.

## Introduction

Multi-task learning (MTL) is a powerful machine learning paradigm that enables the joint modeling of multiple related prediction tasks by sharing information across them. By exploiting task relatedness, MTL can improve generalization, reduce estimation variance^1^, and enhance learning efficiency, particularly in high-dimensional settings where the number of features far exceeds the number of samples. Consequently, MTL has become widely adopted across diverse domains, including biomedicine^2^, computer vision^3^, and natural language processing^4^.

Feature selection plays a central role in high-dimensional data analysis by improving model interpretability and computational efficiency while mitigating the curse of dimensionality^5^. Within the MTL framework, feature selection has been extended to identify predictors that are shared across tasks, for example through sparsity-inducing penalties such as the ℓ₂,₁-norm^6^ or through structured regularization schemes that encode temporal^7^ or task-wise smoothness. While these approaches have demonstrated success in a range of biomedical applications, they are typically developed under the assumption that all tasks share the same type of outcome and loss function, such as purely regression or purely classification settings.

In many real-world applications, however, learning problems naturally involve heterogeneous task types, most commonly a combination of regression and classification objectives. Such mixed-task settings arise, for example, when continuous clinical measurements and binary diagnostic outcomes are modeled jointly^8^, or when the heterogenous outcomes are simultaneously generated from the deep generative system^9^. A fundamental challenge in these scenarios is that regression and classification losses operate on inherently different scales and exhibit distinct optimization behaviors. When naively combined within a single MTL objective, this mismatch can lead to systematic bias in joint optimization, causing certain task types to dominate the learning process and resulting in distorted and unreliable joint feature selection.

Several strategies have been proposed to address task imbalance in mixed-task MTL. A common pragmatic solution is to discretize continuous outcomes^10^, thereby reducing all tasks to a unified classification setting. While this approach forces the balancing of tasks, it inevitably discards valuable ordinal or quantitative information. More recently, dynamic loss-weighting schemes have been introduced, in which task-specific weights are either manually tuned or learned jointly with model parameters based on uncertainty estimates^3^, per-task learning rates^11^, or predictive accuracy^12^. Although these methods are flexible, they are not designed targeting on the joint feature selection particularly in high-dimensional setting.

To address these limitations, we propose MTLComb, a domain-agnostic linear multi-task learning framework that jointly handles regression and classification tasks through an analytical loss-balancing mechanism. MTLComb derives a principled closed-form weighting scheme that aligns the regularization paths of heterogeneous loss functions. This alignment enables unbiased joint feature selection under a shared sparsity-inducing regularization, ensuring that features are selected consistently across tasks of different types. Importantly, the resulting optimization problem remains convex and computationally efficient, preserving interpretability and scalability in high-dimensional settings.

**Fig. 1:**
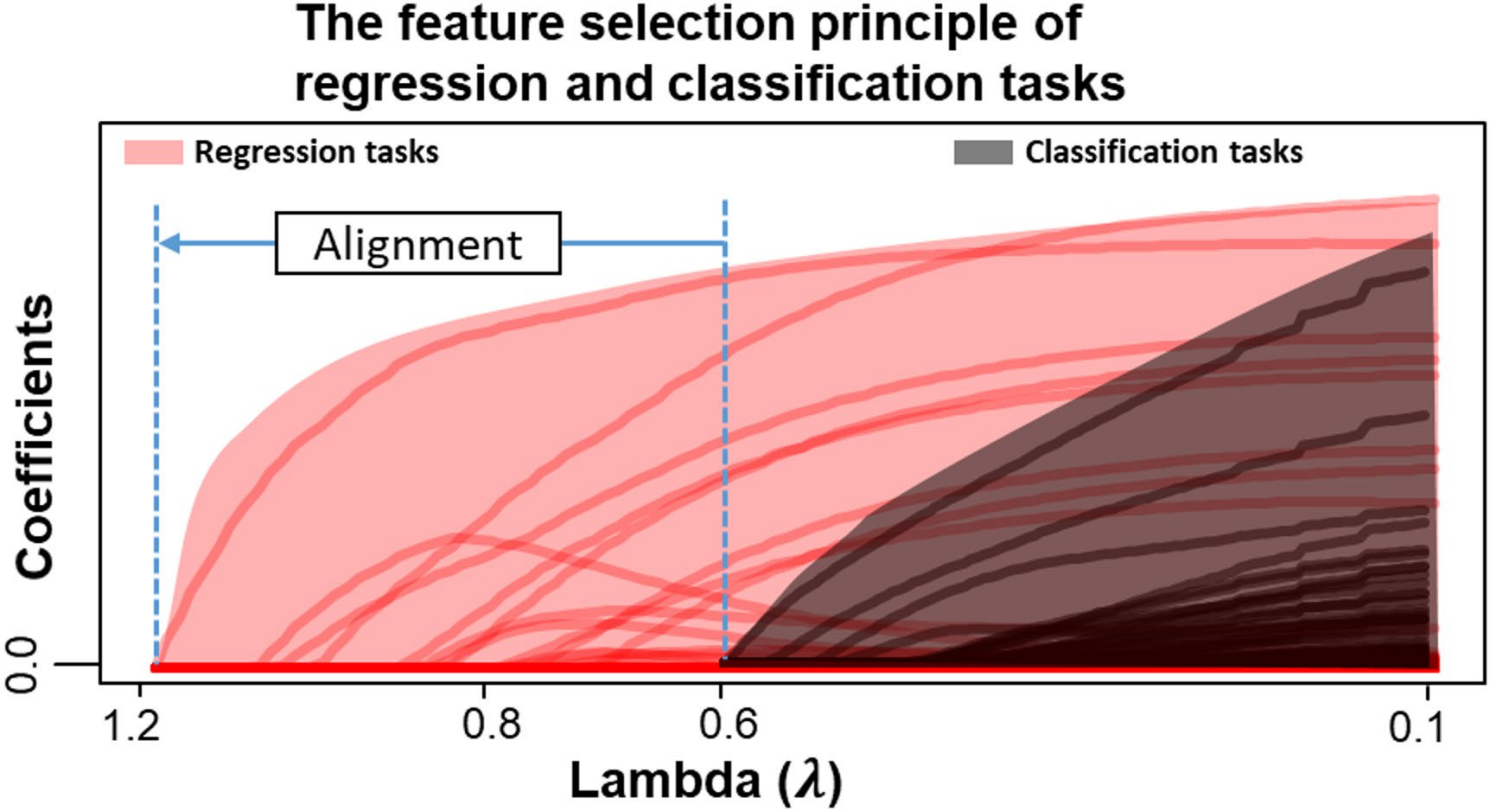
Illustration of MTLComb. The feature selection principle of linear MTL can be quantified as a regularization path^13^. A regularization path refers to a series of models where the coefficients change regarding the variation of a hyperparameter ${\uplambda}$. A large value of ${\uplambda}$ is associated with a fewer number of selected features. Here, the regularization path of regression/classification tasks are shown in red/black color. These paths are not aligned due to the different type of losses, leading to a potentially biased selection of joint features. For example, ${\uplambda}=0.8$ yields 7 features selected for regression tasks but none for classification. MTLComb aims to align the two regularization paths.

As illustrated in Fig. 1, standard regularization paths^13^ for regression and classification tasks are misaligned due to differences in loss magnitudes. For a fixed regularization parameter, regression tasks may select multiple features while classification tasks select none, leading to biased joint feature selection. By analytically rescaling task-specific losses, MTLComb harmonizes these regularization paths, enabling consistent feature selection across heterogeneous tasks using a single regularization parameter.

In two simulation studies, we systematically investigated the performance of MTLComb across varying conditions, including data dimensionality, label imbalance, and the number of tasks, to assess its robustness and domain-agnostic applicability. We further demonstrate the effectiveness of MTLComb in two biomedical case studies. In a sepsis setting, we jointly model diagnostic status and multiple continuous clinical measurements related to metabolic state and kidney function, reflecting shared underlying physiological processes. In a schizophrenia study, we combine age prediction with disease classification to identify age-dependent molecular markers associated with disease risk. In both cases, MTLComb yields balanced feature selection, competitive predictive performance, and improved biological interpretability. The detailed motivation and biological context can be found in the supplementary methods.

## Materials and methods

### Data cohorts and ethical statement

Our retrospective study included six data cohorts, conducting two separate analyses: the sepsis analysis and the schizophrenia analysis. The sepsis analysis involved two cohorts retrieved from electronic medical records used to predict patient risk severity. This analysis, involving human participants, was reviewed and approved by the Medical Ethics Commission II of the Medical Faculty Mannheim, Heidelberg University with the waiver of informed consent (approval numbers: 2016-840R-MA and 2023 − 851).

The discovery cohort for the schizophrenia analysis consisted of the Human Brain Collection Core (HBCC^14^; *n* = 422), comprising genome-wide microarray expression profiles (database of Genotypes and Phenotypes: phs000979.v3.p2). Tissue collection was approved by the Central Nervous System Institutional Review Board (CNS IRB; NCT00001260) and conducted with next-of-kin consent through the Offices of the Chief Medical Examiners in the District of Columbia, Northern Virginia, and Central Virginia. Validation analyses included three independent GEO (Gene Expression Omnibus) cohorts (total *n* = 194): GSE53987^15^, GSE21138^16^, and GSE35977^17^. GSE53987 samples were obtained from the University of Pittsburgh under institutional ethical oversight, including review by the Committee for Oversight of Research and Clinical Training Involving Decedents (CORID). GSE21138 and GSE35977 samples were derived from the Stanley Medical Research Institute Neuropathology Consortium and Array collections, with tissue procurement conducted under standardized institutional protocols and next-of-kin consent. Detailed descriptions and data preprocessing steps are also provided in the supplementary methods.

### Intuition of MTLComb

Figure 1 demonstrates the major challenge of linear MTL with mixed types of tasks for joint feature selection, and serves as the conceptual basis of MTLComb. In regularized models, the feature selection principle can be visualized through the regularization path^13^, which traces how estimated coefficient change with the sparsity-controlling hyperparameter. A higher value of λ is associated with a smaller number of selected features. However, as shown in Fig. 1 when combining regression and classification tasks, the inherent differences in loss function magnitudes cause their regularization paths to be misaligned, leading to a biased joint feature selection. For example, the regression tasks dominate the joint feature selection when${\uplambda}\in\left[\mathrm{0.6,1.2}\right]$, while the classification tasks have no selected features. This imbalance results in a biased joint feature selection process. MTLComb addresses this issue by rescaling the task-specific losses, thereby aligning their regularization paths and enabling consistent feature selection across all tasks using a shared λ.

### Modeling, optimization and algorithms

MTLComb aims at solving the objective

1 $$\min_{W} 2\times Z(W) + 0.5\times R(W) + \lambda\|W\|_{2,1} + \alpha\|WG\|_2^2 + \beta\|W\|_2^2$$

where

$$Z(W) = \sum_{i=1}^{c} \frac{1}{N_i}\log\left(1+\exp\left(-Y^{(i)}X^{(i)}w^{(i)}\right)\right)$$

$$R(W)=\sum_{i=c+1}^{t}\frac{1}{{N}_{i}}{\left|\right|{Y}^{\left(i\right)}-{X}^{\left(i\right)}{w}^{\left(i\right)}\left|\right|}_{2}^{2},$$

$$W=\left[{w}^{\left(1\right)}\cdots{w}^{\left(c\right)}\dots{w}^{\left(t\right)}\right]$$

and $G=\mathrm{d}\mathrm{i}\mathrm{a}\mathrm{g}\left(t\right)-\frac{1}{t}{\mathbf{1}}_{t}{\mathbf{1}}_{t}^{T}$, with ${\left|\right|.\left|\right|}_{2}$denoting the Euclidean norm, $\mathrm{d}\mathrm{i}\mathrm{a}\mathrm{g}(.)$ denoting the operator to construct a diagnal matrix given a constant, ${\mathbf{1}}_{t}$ denoting a t-dimensional column vector with values of one.

$\mathrm{H}\mathrm{e}\mathrm{r}\mathrm{e},\mathrm{Z}\left(W\right)$ is the logit loss to fit the classification tasks, and $\mathrm{R}\left(W\right)$ is the least-square loss to fit the regression tasks. $X=\left\{{X}^{\left(i\right)}\in{\mathbb{R}}^{{N}_{i}\times p}:i\in\left\{1,\dots,c,\dots t\right\}\right\},$ refers to the feature matrices of $t$ tasks, where $p$ features are consistent across tasks. $Y=\left\{{Y}^{\left(i\right)}\in{\mathbb{R}}^{{N}_{i}\times1}:i\in\left\{1,\dots,c,\dots t\right\}\right\}$ describes the outcome lists associated with $c$classification and $t-c$ regression tasks. $W\in{\mathbb{R}}^{p\times t}$ is the coefficient matrix that needs to be estimated, where each column ${w}^{\left(i\right)}\in{\mathbb{R}}^{p\times1}$represents the $p$ coefficients of each task$i$, and each row ${w}_{\left(j\right)}\in{\mathbb{R}}^{1\times\mathrm{t}}$ consists of the coefficients of feature $j$. ${\left|\right|W\left|\right|}_{\mathrm{2,1}}=\sum_{j=1}^{p}{\left|\right|{w}_{\left(j\right)}\left|\right|}_{2}$ is a sparse penalty term to promote the joint feature selection^6^.${\left|\right|WG\left|\right|}_{2}^{2}$ is the mean-regularized term^18^ to promote the similarity of the cross-task coefficients. ${\left|\right|W\left|\right|}_{2}^{2}$ aims to select correlated features and stabilize the numerical solutions^19^. $\left\{\lambda,\alpha,\beta\right\}$ is the set of hyperparameters, which control the strengths of the penalties. Here, $\lambda$ is selected by cross-validation, while α and β are selected by the user as constant priors. We weigh $\mathrm{Z}\left(W\right)$ by 2 and $\mathrm{R}\left(W\right)$ by 0.5. This simple weighting scheme makes the regularization paths consistent.

#### The loss weighting scheme

This weighting scheme is motivated by the observation that the gradient of the least-square loss is four times larger than the gradient of the logit loss evaluated at a parameter where the subgradient equals zero. Consequently, scaling the logit loss by a constant factor four times larger than that used for the regression loss aligns the regularization paths for these mixed-types tasks. We use weighting constants of 2 for the logit loss and 0.5 for the regression loss. A detailed derivation of this weighting scheme is provided in the supplementary methods.

To solve the objective in (1), we adopt the accelerated proximal gradient descent method to approximate the solution^20^, which features a “state-of-the-art” convergence rate of $O\left(1/{k}^{2}\right)$, where k is the number of iterations of the algorithm. The derivation of the optimization procedures and the relevant algorithms are summarized in the supplementary methods.

#### Regularization path estimation

The regularization path, exemplified by the Lasso^13^, illustrates the feature selection principle. MTLComb’s central function is to estimate the complete regularization path, representing a series of models indexed by a sequence of $\lambda$ (a spectrum of sparsity levels). Accurately determining the $\lambda$ sequence is crucial to capture the highest likelihood while avoiding unnecessary explorations. Inspired by glmnet^19^, we estimated the $\lambda$ sequence from the data in three steps. First, we estimate the largest $\lambda$ (referred to as ${\lambda}_{\mathrm{m}\mathrm{a}\mathrm{x}}$) in the sequence, leading to nearly zero coefficients. Second, we calculate the smallest $\lambda$ in the sequence via ${\lambda}_{\mathrm{r}\mathrm{a}\mathrm{t}\mathrm{i}\mathrm{o}}\times{\lambda}_{\mathrm{m}\mathrm{a}\mathrm{x}}$, e.g. with ${\lambda}_{\mathrm{r}\mathrm{a}\mathrm{t}\mathrm{i}\mathrm{o}}=0.01$. Third, we interpolate the entire sequence on the log scale. Calculating ${\lambda}_{\mathrm{m}\mathrm{a}\mathrm{x}}$ with mixed losses poses a challenge, as the optimal estimate for the least squares loss is incompatible with that for the logit loss, as shown in Fig. 1. MTLComb can evaluate a consistent ${\lambda}_{\mathrm{m}\mathrm{a}\mathrm{x}}$ for the mixed losses due to the loss weighting scheme. The detailed algorithm for estimating the regularization path can be found in the supplementary methods.

### Simulation data analysis

In this section, we conducted two simulation-based analyses to quantify the performance of MTLComb under conditions of high data dimensionality and label imbalance in classification tasks. For both analyses, we evaluated model performance using two consistent metrics: prediction accuracy, quantified by the (pseudo-)explained variance, and feature recovery rate, defined as the accuracy of identifying ground-truth features. The detailed construction protocols for the simulation datasets used in both analyses are provided in the Supplementary Methods.

As a primary baseline, we included MTLBin, a conventional multi-task classification approach that binarizes continuous outcomes using their median values. This binarization enables balanced multi-task classification and joint feature selection using standard classification frameworks. However, this procedure inevitably discards ordinal information from the original outcomes and may hinder learning efficiency. Previous work^10^ has adopted MTLBin for jointly selecting features that influence multiple indicators of community health status, where outcomes such as “life expectancy” and “self-rated health status” were binarized to facilitate balanced model training and feature selection.

#### Analysis 1: Impact of data dimensionality

The first analysis aimed to assess the prediction and feature selection performance of MTLComb across settings ranging from low to high data dimensionality. Specifically, we varied the ratio of $\frac{\mathrm{s}\mathrm{u}\mathrm{b}\mathrm{j}\mathrm{e}\mathrm{c}\mathrm{t}\mathrm{n}\mathrm{u}\mathrm{m}\mathrm{b}\mathrm{e}\mathrm{r}}{\mathrm{f}\mathrm{e}\mathrm{a}\mathrm{t}\mathrm{u}\mathrm{r}\mathrm{e}\mathrm{n}\mathrm{u}\mathrm{m}\mathrm{b}\mathrm{e}\mathrm{r}}$ from 0.1 to 0.8, thereby simulating increasingly high-dimensional learning scenarios. For comparison, eight methods were included: MTLComb, MTLBin, and meta-analyses of individual machine-learning approaches, including lasso, ridge regression, random forest, and support vector machines (SVMs) with linear, radial basis function, and polynomial kernels. In the meta-analysis framework, models were trained independently for each task, and their results were subsequently aggregated for prediction and biomarker identification. Detailed methodological specifications for this analysis are provided in the Supplementary Methods.

#### Analysis 2: Impact of label imbalance

The second analysis evaluated the robustness of MTLComb under varying degrees of label imbalance. The imbalance ratio was varied from 0 to 0.5, corresponding to the proportion of positive labels P(Y = 1). For each fixed imbalance ratio, the number of tasks was further varied from 4 to 20, enabling systematic investigation of how cross-task information sharing influences performance under different imbalance conditions. MTLBin was selected as the sole baseline method for this experiment. The detailed setup and parameter configurations for this analysis are described in the Supplementary Methods.

### Real data analysis

#### Prediction of sepsis

MTLComb is trained on clinical features to predict four outcomes: diagnosis (classification task) and the measurements (regression tasks) of lactate, urea, and creatinine. These regression tasks provide insights into the dynamics of the metabolic status and kidney function at sepsis onset. For comparison, we applied MTLBin and common machine learning (ML) methods as baselines.

Two cohorts are included in the analysis, where one serves as the training cohort and the other as the test cohort, and vice versa. This allows for cross-cohort prediction performance evaluation and biomarker reproducibility assessment. AUC is used as the metric for prediction performance. To quantify biomarker reproducibility, we compared the two models (one trained for one cohort) of each approach and count the number of overlapping features selected from the top 10 features. For MTLComb, only the classification model is used for testing. Comparison methods include MTLBin, Lasso^19^, ridge regression^19^, random forest^21^, and SVM^22^.

#### Prediction of schizophrenia

We aimed to identify aging-dependent genes associated with schizophrenia through two defined prediction tasks: predicting the diagnosis of schizophrenia (binary outcome) and predicting the age of subjects (continuous outcome). Given that MTLComb is designed specifically to capture age-dependent risk patterns, our expectation was not to achieve superior prediction performance compared to machine learning methods which have no such restrictions. Hence, this analysis does not focus on prediction accuracy comparisons. Instead, the investigation revolves around whether MTLComb can capture gene markers predictive to all tasks and whether these markers can be validated in another cohort.

The analysis involves a discovery cohort and a validation cohort. In the discovery cohort, first, a 10-fold nested cross-validation procedure quantifies prediction performance. Here, regression and classification models are averaged to predict and identify shared markers. AUC is used for diagnosis prediction, and explained variance is calculated for age prediction. To account for sampling variability, the procedure is repeated 10 times, and the results are averaged. Next, the model trained on all subjects of the discovery cohort is validated on the separate validation cohort. Finally, to demonstrate the biological interpretability of our model, we re-trained the MTLComb models on the discovery and validation cohorts separately. The top 500 genes identified by the model were analyzed using the clusterProfiler^23^ software for pathway enrichment analysis. Homogeneity of the selected genes is compared with other machine learning approaches.

## Results

### Simulation data analysis

#### Analysis 1: Impact of data dimensionality

**Fig. 2:**
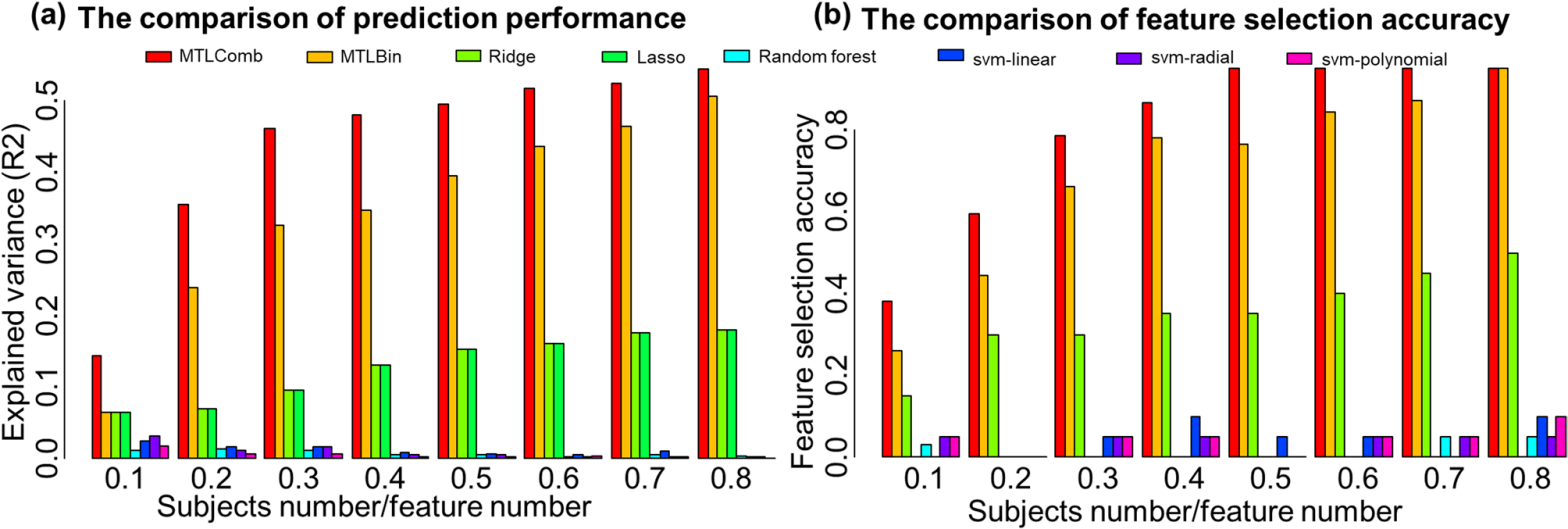
Results of simulation data analysis. **a** The comparison of prediction performance. The superior explained variance of MTL approaches compared to those of ML approaches demonstrates the utility of MTL. With increasing dimensionality, accurate prediction becomes challenging for every algorithm. MTLComb outperforms other methods. **b** The comparison of joint feature selection accuracy. Increasing the dimensionality makes the feature selection more challenging for all algorithms. MTL approaches outperforms meta-analysis. Among MTL approaches, MTLComb outperforms MTLBin, particularly for high dimensional settings.

Figure 2 presents a comprehensive performance comparison across varying data dimensionalities using simulated datasets. Figure 2(a) summarizes prediction performance. The consistently higher explained variance achieved by multi-task learning (MTL) approaches, compared with single-task machine-learning (ML) methods, highlights the advantage of joint modeling. As data dimensionality increases, prediction becomes more challenging for all algorithms. Nevertheless, MTLComb consistently achieves the best performance, followed by MTLBin, demonstrating its superior ability to leverage shared information across tasks.

Figure 2 (b) reports feature selection accuracy under increasing dimensionality. As expected, feature recovery becomes progressively more difficult as dimensionality increases for all methods. However, MTL-based approaches markedly outperform meta-analysis strategies across all settings. Among them, MTLComb consistently yields the highest feature selection accuracy, particularly in high-dimensional regimes, underscoring its effectiveness in extracting relevant signals under challenging conditions.

#### Analysis 2: Impact of label imbalance

**Fig. 3:**
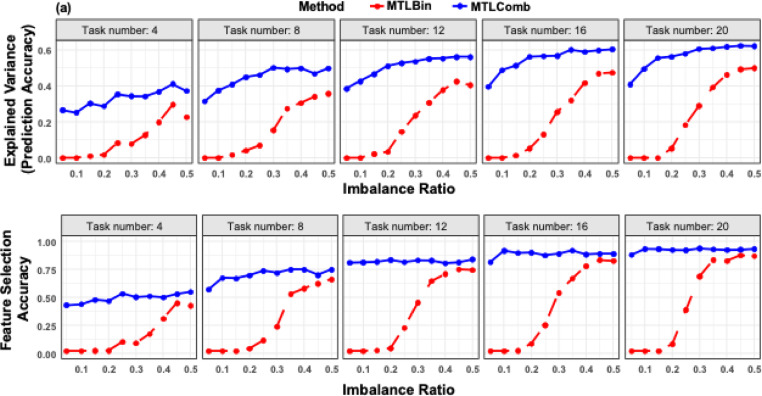
Results of label imbalance data analysis using simulation data. **a** Comparison of prediction performance across varying task numbers and imbalance ratios. MTLComb consistently outperforms MTLBin for all combinations of task numbers and imbalance ratios. For a fixed number of tasks, the performance advantage of MTLComb is more pronounced under severe imbalance (e.g., ratio = 5%). As the number of tasks increases, MTLComb shows consistent performance gains across all imbalance settings, whereas MTLBin exhibits little to no improvement under severe imbalance. **b** Comparison of feature selection accuracy. The overall trends mirror those observed in panel (a), with MTLComb achieving superior and more robust feature recovery across imbalance conditions and task numbers.

Figure 3 compares the performance of MTLComb and MTLBin across varying label imbalance ratios and numbers of tasks. Figure 3(a) shows that MTLComb outperforms MTLBin in prediction accuracy across all combinations of imbalance ratios and task numbers. Notably, the performance advantage of MTLComb becomes more pronounced as the imbalance ratio decreases. When the label imbalance is mild (e.g., ratio = 50%), both methods benefit from increasing the number of tasks. However, under severe imbalance (e.g., 5%), only MTLComb continues to gain from additional tasks. For example, the explained variance of MTLComb increases from approximately 28% to 40%, whereas MTLBin remains at around 1%. These results highlight the robustness of MTLComb under extreme label imbalance.

Figure 3(b) presents feature selection accuracy under the same experimental conditions. Similar to prediction performance, feature selection accuracy improves for both methods as the imbalance decreases. Across all fixed imbalance ratios and task numbers, MTLComb consistently outperforms MTLBin. Importantly, for a given number of tasks, the feature selection accuracy of MTLComb remains relatively stable across different imbalance ratios, further demonstrating its robustness to label imbalance. While increasing the number of tasks improves feature selection for both methods, MTLBin shows limited gains under severe imbalance (e.g., ratio = 5%), indicating its sensitivity to skewed labels. In contrast, MTLComb achieves comparable improvements regardless of imbalance severity, suggesting that it can more effectively exploit cross-task information for feature selection in imbalanced settings.

### Real data analysis

#### Case study 1: Prediction of sepsis

The prediction results are presented in Table 1, where, in general, cohort 1 (training in cohort 2) exhibited better prediction performance than cohort 2 (training in cohort 1), potentially attributable to the more recent data from this cohort (cohort 2) with improved data quality. On average, MTLComb and Ridge regression achieved a similar prediction performance (average AUC ≈ 0.73). MTLComb outperformed Ridge on the prediction of cohort 1, while Ridge showed superior prediction performance in cohort 2. MTLBin was the second most accurate method.

In terms of model interpretability, we compared the model similarity and reproducibility of two representative methods—MTLComb and Ridge. Table 2 illustrates that MTLComb models, trained on independent cohorts, exhibited higher similarity (*r* = 0.7) compared to Ridge regression (*r* = 0.41), highlighting the model stability against cross-cohort heterogeneity. On average, MTLComb identified four reproducible features, whereas Ridge regression yielded 1.2 (average over 10 repetitions), suggesting that MTLComb could pinpoint more reproducible features when applied to an unknown cohort.

Moreover, to demonstrate the biological relevance of MTLComb to the onset of sepsis, we tested the association between predicted scores and kidney function, as well as metabolic state measured on sepsis onset. As shown in Table 2, the MTLComb score demonstrated a higher association than Ridge, indicating that MTLComb could more accurately capture biologically sepsis-relevant associations.

Finally, we list the four features and coefficients selected by MTLComb: ‘SAPSII’ (coefficient: 0.036), ‘SOFA total score’ (coefficient: 0.038), ‘SIRS average λ’^24^ (see supplementary methods for more detail, coefficient: 0.03), and ‘SOFA cardiovascular score’ (coefficient = 0.029). The positive coefficients suggest that all these features were associated with an increment in sepsis risk. This is consistent with our expectation, as the SAPSII score summarizes 17 variables and was developed for the estimation of death risk on ICU admission^25^. The SOFA score represents a concurrent evaluation of organ dysfunction in 6 organ systems^26^ and is an essential part of the current sepsis definition (sepsis-3^27^). A univariable association of the intensity of SIRS with sepsis risk for SIRS average λ has been shown in polytrauma patients^24^. With the exception of SOFA subscore for cardiovascular dysfuntion, the selected features all represent summary scores in which multiple parameters are assessed repeatedly, thereby likely representing patient state more efficiently than features assessed only once. Both SAPSII and SOFA are derived from the most extreme values (maximum or minimum) in the preceding 24 hours, and SIRS average λ reflects the intensity of systemic inflammation over 24 hours. Surprisingly both the total number of points (SOFA total score) as well as the score component reflecting the cardiovascular system were selected, underscoring the importance of circulatory failure for estimation of subsequent sepsis risk and concurrent renal function in polytrauma patients.

#### Case study 2: Prediction of schizophrenia

In the discovery data analysis, a 10-fold nested cross-validation indicates significant predictions for age (t = 2.833, *p* = 0.005, r2 = 0.024) and diagnosis (t = 3.1, *p* = 0.0018, AUC = 0.64) on unseen subjects in the discovery cohort. Subsequently, the model trained using all samples in the discovery cohort was tested on the validation cohort, yielding significant results for age prediction (t = 3.06, *p* = 0.0029, r2 = 0.084) and schizophrenia diagnosis association (t = 4.1, *p* = 4.4e-05, AUC = 0.71).

 Pathway analysis revealed 13 significantly enriched pathways, detailed in Table S4. Notably, several pathways exhibit strong associations with schizophrenia and aging. For instance, voltage-gated channel activity (GO:0022832, FDR = 0.0031), chemical synaptic transmission (GO:0007268, FDR = 0.026), trans-synaptic signaling (GO:0099537, FDR = 0.026), and synaptic signaling (GO:0099536, FDR = 0.034) have all been implicated in the development of schizophrenia and the aging process through their roles in synaptic plasticity^28,29^.

**Fig. 4:**
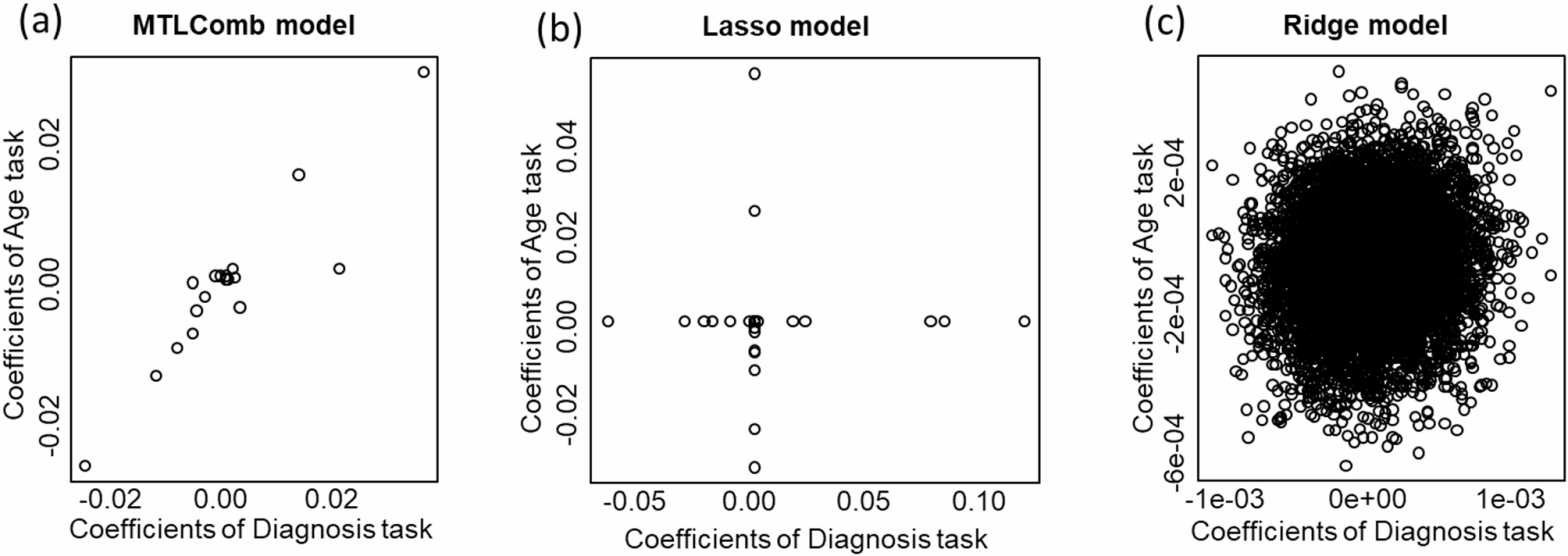
Homogenous marker selection for age and diagnosis prediction in the schizophrenia setting. The models are trained on the discovery cohort. MTLComb is trained simultaneously on two tasks. For lasso and ridge regression, the model is trained for each outcome individually. The coefficients of both prediction tasks are shown for the MTLComb (**a**), lasso (**b**) and ridge (**c**) methods.

To demonstrate that MTLComb allowed the selection of a more homogenous set of markers, the coefficients of the MTLComb model for both age- and diagnosis-prediction tasks are shown in Fig. 4(a). For comparison, ridge and lasso models, trained individually for each outcome, are also plotted in Fig. 4 (c) and (b). Specifically, In Fig. 4(b), individual lasso models select very different markers for each task. In Fig. 4 (c), the ridge regression models tend to consider the risk patterns of all features, individually predictive for each task. Therefore, due to complex correlations among genes in brain cohorts, many markers exhibited different behaviors in different tasks, complicating interpretation. In contrast, MTLComb identified the same markers with similarly predictive behaviors for all outcomes, as shown in Fig. 4(a), which may index a shared molecular mechanism with higher likelihood.

## Discussion

In this manuscript, we introduced MTLComb, a novel MTL algorithm designed to identify shared biomarkers across mixed regression and classification tasks. At its core, MTLComb employs a principled loss-weighting scheme that derives analytically optimal weights across heterogeneous loss functions, thereby alleviating task imbalance. Complementing this formulation, we developed an efficient optimization strategy, along with a dedicated training protocol and hyperparameter tuning procedure. Together, these components enable MTLComb to achieve state-of-the-art convergence rates, making it well suited for large-scale applications. We demonstrated the effectiveness of MTLComb through two comprehensive simulation studies, in which it consistently outperformed competing approaches across varying data dimensionalities, label imbalance ratios, and numbers of tasks. These results indicate that MTLComb functions as an application-agnostic framework, imposing zero domain-specific assumptions and allowing flexible adaptation to diverse applications settings. In the subsequent real data analysis of sepsis, MTLComb exhibited competitive prediction performance, increased model stability, higher marker selection reproducibility, and greater biological interpretability in the sepsis context. This positions MTLComb as a data-integrative tool capable of aggregating subtle risk signals from heterogeneous clinical and biological datasets into a discernible and reproducible pattern. In the schizophrenia analysis, MTLComb successfully captured homogeneous gene markers predictive in both age- and diagnosis-prediction tasks, validated in an independent cohort. This suggests the potential of MTLComb to stratify age-dependent schizophrenia risk, offering novel applications in precision medicine. Here, we identified several synaptic signaling pathways that had previously been associated with both schizophrenia, as well as aging, likely due to their relevance for synaptic plasticity. For example, voltage-gated channel activity has been linked to schizophrenia due to its mediation of intracellular Ca2 + influx, which alters neuronal excitability and synaptic plasticity^30^. Interestingly, the regulation of Ca2 + also changes with aging, disrupting Ca2 + homeostatic processes and affecting the transmission of information across brain systems^31^. These transmission properties are crucial for the induction of synaptic plasticity. Our analysis supports the hypothesis that synaptic plasticity may represent a common pathway of relevance for both schizophrenia^32^ and aging^33^. However, due to potential confounding factors such as medication effects in brain-expression, these findings should be interpreted with caution. The ability to identify signatures of shared relevance for different phenotypes may make MTLComb useful for comorbidity analysis, as has been previously performed using regression-based MTL^34^.

It is important to note that MTLComb has several limitations. With a regularization approach based on the linear model, MTLComb has favorable properties for analysis of high-dimensional problems but yields limited improvements in low-dimensional scenarios, as depicted in Fig. 2 (b). This observation is matched in the sepsis analysis presented in Table 1, where MTLComb demonstrated a prediction performance similar to that of ridge regression. Nevertheless, as indicated in Table 2, the utility of model interpretability and biological plausibility is still evident in the low-dimensional context despite the comparable prediction performance. This could be attributed to the inherent confounding in clinical data^35^, where the confounding effect could be mitigated by learning information from multiple tasks using MTL. This aligns with our previous investigation^1^ highlighting MTL’s robustness against the cross-cohort and sampling variability when applied to heterogeneous cohorts. Second, although MTLComb greatly harmonized the feature selection principle of different types of tasks, there are still differences in the magnitude of the coefficients. An appropriate z-score standardization could minimize this problem, but further research is needed to solve this problem.

Future work will broaden MTLComb’s flexibility in two directions. First, we can integrate additional loss functions—such as a Poisson regression term^36^—to handle count outcomes (e.g. ICU-stay length²³) alongside our existing regression and classification objectives. Second, although our current ℓ₂,₁–norm penalty enables joint feature selection, it does not always yield the level of sparsity that some applications demand. To address this, we plan to incorporate a more aggressive ℓ₂,₀–norm approximation: since directly solving an ℓ₂,₀ penalty is NP-hard^37^, we will adopt the projection-based heuristic strategy^38^, which efficiently approximates ℓ₂,₀ solutions in practice.

Although the two real-world case studies presented in this work are drawn from biomedical applications, the formulation of MTLComb is inherently domain-agnostic. Its loss-weighting scheme relies solely on the mathematical properties of logistic and least-squares losses, together with regularization path estimation, rather than on domain-specific assumptions. The joint feature selection mechanism can therefore be applied broadly to settings in which multiple regression and classification tasks share a common feature space. As demonstrated in the simulation studies, MTLComb exhibits robust and systematic performance improvements across a wide range of data dimensionalities, label imbalance ratios, and numbers of tasks.

## Conclusion

In this work, we introduced MTLComb, a novel linear multi-task learning framework that incorporates a principled loss-weighting mechanism to enable balanced joint feature selection across heterogeneous loss functions. Through two simulation studies, we systematically evaluated the behavior of MTLComb under varying data dimensionalities, label imbalance ratios, and numbers of tasks, demonstrating its robustness and domain-agnostic nature. Using two real-world biomedical applications—sepsis outcome prediction and age-dependent schizophrenia risk modeling—we further showed that MTLComb not only matches or exceeds the predictive performance of standard baseline methods but also produces more stable and biologically plausible feature sets. These properties underscore the utility of MTLComb for biomarker discovery and integrative biological analysis.

Beyond biomedicine, we envision MTLComb playing a vital role in any domain characterized by heterogeneous prediction tasks - ranging from multi-modal vision systems to finance and cybersecurity applications where regression and classification objectives coexist. By unifying diverse outcomes under one interpretable, efficient framework, MTLComb opens new opportunities for integrated data analysis, mechanistic insight, and precision decision-making across scientific and engineering disciplines.

**Table 1:** Cross-cohort predictive performance regarding early sepsis prediction. Hyperparameters were determined using 10-fold cross-validation on each training dataset. The final model was trained on the entire training dataset and subsequently evaluated on the independent test dataset. MTLComb and MTLBin are MTL approaches and the rest are machine learning approaches. MTLBin referred to a conventional multi-task classification approach, in which continuous outcomes are converted into binary outcomes. For each testing cohort, the best performance is highlighted and bolded.

Metric	Test ​on cohort	MTLComb	MTLBin ​	Ridge	Lasso	Random forest	SVM-linear ​	SVM-radial ​	SVM-polynomial ​
AUC	1	**0.75**	0.73	0.73	0.70	0.71	0.64	0.71	0.70
	2	0.71​	0.70 ​	**0.73​**	0.68	0.67​	0.65​	0.72	0.69​

**Table 2:** The model interpretability. Model similarity was assessed using the correlation coefficient between coefficient vectors derived from two independent cohorts. Reproducibly selected features are defined as the overlap of the top ten selected features across cohorts. Prediction outcomes (lactate, creatinine, and urine) were measured at patients’ onset time points in the test data.

	MTLComb	Ridge regression
Model similarity	0.70	0.41
The number of reproducibly selected features	4.0	1.2
Predict lactate value (explained variance)	10%	4%
Predict on creatinine (explained variance)	5.4%	1.2%
Predict on urine (explained variance)	15%	5%

## Supplementary Information

Below is the link to the electronic supplementary material.

Supplementary Material 1

## Data Availability

Part of the data presented in this article is publicly accessible, while the remaining data can be made available upon request. For the simulation analysis, data generation details are provided in our GitHub repository, which is specified in the section of code availability. In the schizophrenia analysis, the datasets are publicly available on dbGAP and GEO repositories. Specifically, the discovery data used in this study is obtained from the dbGaP database (accession number: phs000979.v3.p2). The validation data used was obtained from three GEO repositories: GSE53987, GSE21138, and GSE35977. Detailed description of these datasets are listed in the supplementary methods. For the sepsis analysis, data access requests should be directed to the corresponding author, Verena Schneider-Lindner, at [Verena.Schneider-Lindner@medma.uni-heidelberg.de](mailto: Verena.Schneider-Lindner@medma.uni-heidelberg.de).

## Code availability

https://github.com/transbioZI/MTLComb.
