# Supplementary material for: A multi-task learning approach combining regression and classification tasks for joint feature selection
Source: Sci Rep. 2026 Apr 17;16:12699. doi: 10.1038/s41598-026-43551-3 (PMC13090349; doi:10.1038/s41598-026-43551-3)
Supplement: Supplementary file 1 — Supplementary Material 1 [file 41598_2026_43551_MOESM1_ESM.pdf]

A multi-task learning approach combining regression and classification tasks for joint feature selection

Han Cao[1], Sivanesan Rajan[2,3], Bianka Hahn[4], Ersoy Kocak[2,3], Manuel Brenner[5], Florian Hess[1,6], Roman Schefzik[2,3], Daniel Durstewitz[1,5,6], Georgia Koppe[1,2,3,5], Emanuel Schwarz[2,3]*, Verena Schneider-Lindner[4]*

1 Department of Theoretical Neuroscience, Central Institute of Mental Health, Medical Faculty, Heidelberg University, Germany

2 Department of Psychiatry and Psychotherapy, Central Institute of Mental Health, Medical Faculty, Heidelberg University, Mannheim, Germany

3 Hector Institute for Artificial Intelligence in Psychiatry, Central Institute of Mental Health, Medical Faculty Mannheim, Heidelberg University, Mannheim, Germany

4 Department of Anesthesiology and Surgical Intensive Care Medicine, Medical Faculty Mannheim, Heidelberg University, Theodor-Kutzer-Ufer 1-3, 68167, Mannheim, Germany

5 Interdisciplinary Center for Scientific Computing, Heidelberg University, J5, 68159 Mannheim, Germany

6 Faculty of Physics and Astronomy, Heidelberg University, J5, 68159 Mannheim, Germany

Outline

- Modeling, optimization and algorithms
- The derivation of losses weighting scheme
- Simulation data analyses
  - Analysis 1: Impact of data dimensionality
  - Analysis 2: Impact of label imbalance
- Case study 1: Prediction of sepsis
  - Motivation
  - Data cohorts
  - Data preprocessing
- Case study 2: Prediction of schizophrenia
  - Motivation
  - Data cohorts
  - Data preprocessing

Modeling, optimization and algorithms

$$\min_{W} 2 \times \mathrm{Z}(W) + 0.5 \times \mathrm{R(W)} + \lambda||W||_{2,1} + \alpha||WG||_2^2 + \beta||W||_2^2, \quad (1)$$

where

$\mathrm{Z}(W) = \sum_{i=1}^{c} \frac{1}{N_i} \log(1 + e^{-Y^{(i)}(X^{(i)}w^{(i)})}),$

$\mathrm{R}(W) = \sum_{i=c+1}^{t} \frac{1}{N_i} ||Y^{(i)} - X^{(i)}w^{(i)}||_2^2,$

$W = \left[w^{(1)} \cdots w^{(c)} \dots w^{(t)}\right]$

and $G = \mathrm{diag}(t) - \frac{1}{t}\mathbf{1}_t\mathbf{1}_t^T$, with $||.||_2$ denoting the Euclidean norm, $\mathrm{diag}(.)$ denoting the operator to construct a diagnal matrix given a constant, $\mathbf{1}_t$ denoting a t-dimensional column vector with values of one.

Here, $\mathrm{Z}(W)$ is the logit loss to fit the classification tasks, and $\mathrm{R}(W)$ is the least-square loss to fit the regression tasks. $X = \left\{X^{(i)} \in \mathbb{R}^{N_i \times p}: i \in \{1, \dots, c, \dots t\}\right\}$, refers to the feature matrices of t tasks, where p features are consistent across tasks. $Y = \left\{Y^{(i)} \in \mathbb{R}^{N_i \times 1}: i \in \{1, \dots, c, \dots t\}\right\}$ describes the outcome lists associated with c classification and $t - c$ regression tasks. $W \in \mathbb{R}^{p \times t}$ is the coefficient matrix that needs to be estimated, where each column $w^{(i)} \in \mathbb{R}^{\mathrm{p} \times 1}$represents the coefficient vector of each task i, and each row $w_{(j)} \in \mathbb{R}^{1 \times \mathrm{t}}$ consists of the coefficients of feature j. $||W||_{2,1} = \sum_{j=1}^{p} ||w_{(j)}||_2$ is a sparse penalty term to promote the joint feature selection[1]. $||WG||_2^2$ is the mean-regularized term[2,3] to promote the similarity of the cross-task coefficients. $||W||_2^2$ aims to select the correlated features and stabilize the numerical solutions[4]. $\{\lambda, \alpha, \beta\}$ is the set of hyperparameters, which control the strengths of the penalties. Here, λ is selected by cross-validation, while α and β are selected by the user as constant priors. We weight $\mathrm{Z}(W)$ by 2 and $\mathrm{R}(W)$ by 0.5. This simple weighting scheme makes the regularization paths consistent.

The MTLComb for integrating regression and classification tasks with joint feature selection is formulated as (1). This problem is not easy to solve due to the non-smooth penalty. To solve it efficiently, we adopt the accelerated proximal gradient descent method to approximate the solution, yielding state-of-the-art efficiency.

Let $F(W) = 2 \times \mathrm{Z}(W) + 0.5 \times \mathrm{R(W)} + \alpha||WG||_2^2 + \beta||W||_2^2$ and $\Omega(W) = ||W||_{2,1}$, the formulation (1) is re-written as (2),

$$\min_{W} F(W) + \lambda\Omega(W) \quad (2)$$

$F(W)$ is a smooth and convex function about W, and $\Omega(W)$ is the non-smooth and convex. Assume the Lipschitz constant of $F(W)$ is L, the key step is to solve the following subproblem (3) in each iteration,

$$W_{i+1} = \arg\min_{y} F(W_i) + \langle \nabla F(W_i), \mathrm{y} - W_i \rangle + \frac{L}{2} \left|\left|y - W_i\right|\right|_2^2 + \lambda\Omega(y) \tag{3}$$

Problem (3) is the second-order approximation of $F(.)$ given the current standing point W_i. After re-organization, we have an equivalent form (4),

$$W_{i+1} = \arg\min_{y} \frac{L}{2}\left(y - \left(W_i - \frac{\nabla F(W_i)}{L}\right)\right)^2 + \lambda||y||_{2,1} \tag{4}$$

Let $w^{(j)}{}_i \in \mathbb{R}^{1\times t}$ is the jth row of W_i, problem (4) can be divided into p independent small problems to solve. Such a "divide and conquer" strategy greatly reduces the complexity of the problem. Each small problem took the form (5),

$$w^{(j)}{}_{i+1} = \arg\min_{y} \frac{L}{2}\left(y - \widetilde{w}^{(j)}{}_i\right)^2 + \lambda\sqrt{\left|\left|y^{(j)}\right|\right|^2} \tag{5}$$

where

$$\widetilde{w}^{(j)}{}_i = w^{(j)}{}_i - \frac{\nabla F(w_i)^{(j)}}{L}$$

This problem (5) can be solved analytically

$$w^{(j)}{}_{i+1} = \left(1 - \frac{\lambda/_L}{\max\left\{\left|\left|\widetilde{w}^{(j)}{}_i\right|\right|_2, \lambda/_L\right\}}\right)\widetilde{w}^{(j)}{}_i \tag{6}$$

Since the Lipschitz constant L is usually unknown, we estimated it using the Ajimo-Goldstein line search[5] in MTLComb. Since the objective of MTLComb is convex and non-smooth, we accelerate the entire procedure with the momentum methods. Here, we applied Nesterov's acceleration approach [6-8]. For each iteration i, a search point was quantified by weighing the estimates from the previous two iterations,

$$\mathrm{s_i} = \frac{\alpha_{\mathrm{i}-1}}{\alpha_{\mathrm{i}}}\mathrm{w_i} + \frac{1-\alpha_{\mathrm{i}-1}}{\alpha_{\mathrm{i}}}\mathrm{w_{i-1}} \tag{7}$$

The search point was subsequently sent to (6) to update the solution. The procedure will repeat until the solution converges to the required precision. The entire algorithm integrating all these techniques is summarized in **Algorithm 1.**

**Algorithm 1** The solver of MTLComb
**Input**: $\lambda > 0, L_0 > 0, W_0, maxIter > 0$<br>**Output**: W_{i+1}<br>1: Initialize $W_1 = W_0$, $\alpha_{-1} = \alpha_0 = 0$, and $L = L_0$<br>2: **for** $i = 1$ to $maxIter$ **do**

3:	Set $\mathrm{S_i} = \mathrm{W_i} + \frac{\alpha_{i-1}-1}{\alpha_i}(\mathrm{W_i} - \mathrm{W_{i-1}})$	
4:	Find the smallest $L \in \{L_{i-1}, 2L_{i-1}, 4L_{i-1}, 16L_{i-1}, \ldots\}$ such that $$F(\mathrm{S_i}) + \langle \nabla F(\mathrm{S_i}), W_{i+1} - \mathrm{S_i}\rangle + \frac{L}{2}\left\|W_{i+1} - W_i\right\|_2^2 \geq F(W_{i+1}),$$ where W_{i+1} is quantified according to (4) and (5)	
5:	Set $L_i = L$, and $\alpha_{i+1} = \frac{1+\sqrt{1+4\alpha_i^2}}{2}$	
6:	If the termination rule is satisfied, **return**	
7: **end for**		

The derivation of losses weighting scheme

In this section, we will show that scaling $\mathrm{Z}(W)$ by $4C$ and $\mathrm{R(W)}$ by C where $C > 0$, the regularization path is aligned with a consistent form of the largest λ ($\lambda_{\max}$).

Let m be the index of a classification task, j be the index of a feature. Then w_{jm} is an element of the coefficient matrix W_c of classification tasks. $w_{j,}$ refers to the jth row of W_c, representing the coefficients of feature j. $w_{,m}$ refers to the ith column of W_c, representing the coefficients of task m. N_m refers to the number of subjects of task m. t is th number of all tasks. d is the number of classification tasks. The total loss function becomes:

$$\min_W 4\mathrm{C} \times \mathrm{Z}(W) + \mathrm{C} \times \mathrm{R(W)} + \lambda||W||_{2,1} + \alpha||WG||_2^2 + \beta||W||_2^2,$$

The subgradient regarding the coefficient w_{jm} took the form (8),

$$\partial_{w_{jm}} = \frac{4C}{N_m}\sum_{i=1}^{N_m}\frac{-y_i^{(m)}x_{ij}^{(m)}}{1+\exp\left(y_i^{(m)}x_i^{(m)}w_{,m}\right)} + 2\beta w_{jm} + 2\alpha GGw_{j,}{}^T + \lambda\frac{w_{jm}}{||w_{j,}||_2} \quad (8)$$

Let $A_m = \frac{4C}{N_m}\sum_{i=1}^{N_m}\frac{-y_i^{(m)}x_{ij}^{(m)}}{1+\exp\left(y_i^{(m)}x_i^{(m)}w_{,m}\right)} + 2\beta w_{jm} + 2\alpha GGw_{j,}{}^T$

Then the subgradient of the coefficients of feature j, $w_{j,}$ is

$$\partial_{w_{j,}} = \begin{bmatrix} A_1 + \lambda\frac{w_{j1}}{||w_{j,}||_2} \\ \vdots \\ A_m + \lambda\frac{w_{jm}}{||w_{j,}||_2} \\ \vdots \\ A_d + \lambda\frac{w_{jc}}{||w_{j,}||_2} \end{bmatrix} \quad (9)$$

Let $\partial_{w_{j,}} = 0$ and $W_c = \mathbf{0}$ where the nonsmoothness occupied, we have

$$\partial_{w_{j,}} = \begin{bmatrix} A_1 + \lambda v_1 \\ \vdots \\ A_m + \lambda v_m \\ \vdots \\ A_d + \lambda v_d \end{bmatrix} = \mathbf{0} \tag{10}$$

Where $v = \{x \in R^d: ||x||_2 \leq 1\}$, and $A_m = -\frac{2C}{N_m}\sum_{i=1}^{N_m} y_i^{(m)} x_{ij}^{(m)}$

We then have a set of simplified equations

$$\frac{2C}{N_1}\sum_{i=1}^{N_1} y_i^{(1)} x_{ij}^{(1)} = \lambda v_1$$
$$\vdots$$
$$\frac{2C}{N_m}\sum_{i=1}^{N_m} y_i^{(m)} x_{ij}^{(m)} = \lambda v_m$$
$$\vdots$$
$$\frac{2C}{N_d}\sum_{i=1}^{N_d} y_i^{(d)} x_{ij}^{(d)} = \lambda v_d$$

Since we assume λ strictly positive, we can calculate λ by taking the square on both sides, summation and root.

$$\lambda = \frac{2C\sqrt{\sum_{m=1}^{d}\left[\frac{\sum_{i=1}^{N_m} y_i^{(m)} x_{ij}^{(m)}}{N_m}\right]^2}}{||v||_2} \tag{11}$$

The smallest λ is obtained when $||v|| = 1$,

$$\lambda^* = \min_{v} \frac{2C\sqrt{\sum_{m=1}^{c}\frac{1}{{N_m}^2}\left[\sum_{i=1}^{N_m} y_i^{(m)} x_{ij}^{(m)}\right]^2}}{||v||_2} = 2C\sqrt{\sum_{m=1}^{d}\left[\frac{\sum_{i=1}^{N_m} y_i^{(m)} x_{ij}^{(m)}}{N_m}\right]^2} \tag{12}$$

Therefore, $\lambda^{*(j)} = 2C\sqrt{\sum_{m=1}^{d}\left[\frac{\sum_{i=1}^{N_m} y_i^{(m)} x_{ij}^{(m)}}{N_m}\right]^2}$ for the feature j of the classification task.

Similarly, for a regression task n, we assume the coefficient w_{jn} an element of the coefficient matrix W_r of regression tasks. $w_{j,}$ refers to the jth row of W_r, representing the coefficients of feature j. $w_{,n}$ refers to the ith column of W_r, representing the coefficients of task n. N_n refers to the number of subjects of task n. t is the number of all tasks. $t - d$ is the number of regression tasks

The subgradient about the coefficient w_{jn} is

$$\partial_{w_{jn}} = \frac{2C}{n_n} {X_{,j}^{(n)}}^T (X^{(n)} w_{,n} - y^{(n)}) + 2\beta w_{jn} + 2\alpha G G {w_{j,}}^T + \lambda \frac{w_{jn}}{||w_{j,}||_2}, \tag{13}$$

After applying the same procedure as for the classification task, we give directly the solution for regression task n, , $\lambda^{*(j)} = 2C\sqrt{\sum_{n=1}^{t-d}\left[\frac{\sum_{i=1}^{N_n} y_i^{(n)} x_{ij}^{(n)}}{N_n}\right]^2}$

It is clear that , $\lambda^{*(j)}$ take the same form for both classification and regression tasks.

In the end, the largest λ ($\lambda_{\max}$) of the total loss (1) took the form after scanning all feature$\lambda_{\max} = \max_{j} 2C\sqrt{\sum_{n=1}^{t}\frac{1}{{N_n}^2}\left[\sum_{i=1}^{N_n} y_i^{(n)} x_{ij}^{(n)}\right]^2}$

For simplicity, in MTLComb, we choose $C = 0.5$ resulting in the final formulation (1)

The entire algorithm for estimating the regularization path is shown in **Algorithm 2**

**Algorithm 2** The estimation of the regularization path
**Input**: $\lambda_1 > \lambda_2 > \cdots > 0$<br>**Output**: W_1, W_2,…<br>1: Initialize $W_0 = p \times t = 0$<br>2: **for** $i = \{1, 2, \dots\}$ **do**<br>3: W_i =**Algorithm 1** ($\lambda = \lambda_i, L_0 = 1, W_0 = W_{i-1}, maxIter = 100$)<br>4: **end for**

Parameter tuning

The optimal λ is determined by k-fold cross-validation (CV) in MTLComb. α and β are determined by the users as prior. The intercept and intercept-free models are both provided to fit normalized and unnormalized data, respectively.

Simulation data analysis

Analysis 1: Impact of data dimensionality

Data generation protocol. We simulated a total of 20 tasks, comprising 10 classification tasks and 10 regression tasks. The number of tasks was fixed at t=20, and the number of features was set to p=500. The sample size for each task was varied as

$$N_m \in \{0.1,0.2,0.3,0.4,0.5,0.6,0.7,0.8\} \times p,$$

thereby generating scenarios ranging from high- to low-dimensional settings.

The coefficient matrix $W \in \mathbb{R}^{p\times t}$ was sampled from a standard normal distribution,

$$W \sim \aleph(0,1).$$

To induce sparsity, 90% of the features were set to zero by enforcing

$$W[(0.1p + 1){:}\,p, :] = 0.$$

For each task m, the feature matrix was generated as

$$X^{(m)} \sim \aleph_{N_m \times p}(0,1),$$

where N_m denotes the sample size of task m.

For regression tasks, outcomes were generated according to

$$Y^{(m)} = X^{(m)} \times W_{:,m} + 0.5 \times \aleph_{N_m \times 1}(0,1).$$

For classification tasks, outcomes were obtained as

$$Y^{(m)} = \left[X^{(m)} \times W_{:,m} + 0.5\aleph_{N_m \times 1}(0,1)\right]_+,$$

where $[\cdot]_+$ denotes the binarization operator. The noise term corresponds to a signal-to-noise ratio in which random noise accounts for 50% of the signal strength.

Machine learning procedure. Eight algorithms were included in this analysis. With the exception of support vector machines (SVMs) and random forests, 5-fold cross-validation was used to select hyperparameters. For multi-task learning approaches (MTLComb and MTLBin), all 20 tasks were trained jointly, and predictions were generated on held-out test datasets to compute explained variance. For single-task machine-learning methods—including Lasso, ridge regression, random forest, and SVMs with different kernels—models were trained independently for each task and evaluated on corresponding test datasets drawn from the same distribution.

To quantify feature selection accuracy, we first averaged the absolute values of the estimated coefficient vectors across all tasks for each method. The top $0.1 \times p$ features were then selected as the model-identified feature set. Feature selection accuracy was defined as

$$\frac{|\text{ Selected features} \cap \text{Ground truth }|}{0.1 \times p}.$$

To account for the sampling variance, the above procedure is repeated 10 times, and the results are averaged for investigation. Detailed implementation and calculations are provided in the accompanying R scripts available in the GitHub repository.

Analysis 2: Impact of label imbalance

Data generation protocol. We simulated a mixed-task setting with t tasks, consisting of an equal number of classification and regression tasks. Specifically, the classification task set was defined as

$$C = \{1,2, \ldots, t/2\},$$

and the regression task set was

$$R = \{1, \ldots, t\} \setminus C$$

The feature dimension was fixed at p=500. For each task, we generated n=100 training samples and an additional n=100 independent test samples.

A shared coefficient matrix $W \in \mathbb{R}^{p \times t}$ was constructed to induce sparsity. Each entry of W was sampled with random sign and random magnitude, where the magnitude is centered around 1:

$$W_{j,m} = \text{sign}(z_{j,m}) \times u_{j,m},\ z_{j,m} \sim \aleph(0,1),\ u_{j,m} \sim \aleph(1,1).$$

To enforce feature-level sparsity, only the first $0.1 \times p$ features were kept as non-zero across tasks, and the remaining coefficients were set to zero:

$$W[(0.1p+1){:}\,p, :] = 0.$$

For each task m, the training and test feature matrices were sampled independently from a standard normal distribution:

$$X^{(m)} \in R^{n \times p}, \quad \tilde{X}^{(m)} \in R^{n \times p}, \quad X_{ij}^{(m)}, \tilde{X}_{ij}^{(m)} \sim N(0,1).$$

For classification tasks ($m \in C$), we first computed latent scores

$$s^{(m)} = X^{(m)} W_{:,m} + \epsilon, \quad \tilde{s}^{(m)} = \tilde{X}^{(m)} W_{:,m} + \tilde{\epsilon},$$

where $\epsilon, \tilde{\epsilon} \sim N(0, \sigma^2 I)$ and σ=noise (set to 0.5 in our experiments). To control the label imbalance, we defined the imbalance ratio ρ=**imbRate**$\in$[0,0.5] as the proportion of the positive class. We then thresholded the latent scores using the (1−ρ)-quantile:

$$\tau^{(m)} = Q_{1-\rho}(s^{(m)}), \quad \tilde{\tau}^{(m)} = Q_{1-\rho}(\tilde{s}^{(m)}),$$

and generated binary labels encoded as $\{-1, +1\}$:

$$Y^{(m)} = \begin{cases} +1 & s^{(m)} > \tau^{(m)} \\ -1 & \text{otherwise} \end{cases}, \quad \tilde{Y}^{(m)} = \begin{cases} +1 & \tilde{s}^{(m)} > \tilde{\tau}^{(m)} \\ -1 & \text{otherwise} \end{cases}$$

When $\rho \leqslant 0$, all labels were set to the negative class; when ρ>0.5, we capped it at 0.5 to avoid generating a majority-positive setting.

For regression tasks ($m \in R$), outcomes were generated using a linear model with Gaussian noise:

$$Y^{(m)} = X^{(m)} \times W_{:,m} + \epsilon, \quad \tilde{Y}^{(m)} = \tilde{X}^{(m)} \times W_{:,m} + \tilde{\epsilon},$$

with the same noise level σ=noise=0.5.

Finally, we standardized features task-wise by z-scoring each column of $X^{(m)}$ and $\tilde{X}^{(m)}$. For regression tasks, we additionally standardized the continuous outcomes $Y(m)$ (task-wise), ensuring comparable scales across tasks.

Machine learning procedure. Multiple simulation datasets were generated by varying the label imbalance ratio

$$\text{imbRate} \in \{0.05, 0.1, 0.15, 0.2, 0.25, 0.3, 0.35, 0.4, 0.45, 0.5\}$$

and and the number of tasks

$$\text{t} \in \{4, 8, 12, 16, 20\}.$$

For each simulated dataset, MTLComb and MTLBin were trained following the same procedures described in the previous section. To account for sampling variability, each experimental setting was repeated 10 times using independently generated datasets, and performance metrics were averaged across repetitions. Detailed implementation and calculations are provided in the accompanying R scripts available in the GitHub repository.

Case study 1: Prediction of sepsis

Motivation

Sepsis is the leading cause of mortality in intensive care units (ICU)[9] and critical illness worldwide[10]. It is caused by the dysregulated host response to infection and organ dysfunction, and has been associated with high risk of complications, e.g., ICU death, longer hospital stays and higher medical costs[11,12]. Early detection of sepsis plays a crucial role in improving patient survival rates[13] and reducing medical expenses. As a response, a research line has emerged to predict various clinical outcomes of sepsis, such as diagnosis[14], ICU mortality[12] and ICU stay[15], leveraging early clinical features — typically referring to patient data upon ICU admission. However, an individual outcome (e.g., diagnosis of sepsis) prediction model may not fully capture the underlying risk patterns associated with sepsis. In this case study, we employ MTLComb to discern sepsis risk factors that can simultaneously predict diagnosis (classification task), kidney function (regression task), and metabolic measurements (regression task). This choice is grounded in the understanding that the changes in metabolic variation and kidney functioning accompanying sepsis onset are driven by the same biological processes. We hypothesize that this multi-task learning formulation will yield a more predictive and interpretable model.

Data cohorts

Two cohorts of polytrauma patients were retrospectively created from the electronic medical records of the surgical ICU of the University Medical Center Mannheim, Germany. Cohort 1 consisted of 242 patients treated between 2006 and 2011[16], and contained 78 sepsis cases and 164 controls with sufficient data for this study. Cohort 2 with 159 patients was treated in the same ICU thereafter, from 2011 to 2016 and contained 56 sepsis cases and 103 controls. Ethical approval for involving cohort 1 and 2 in our study were obtained from the Medical Ethics Commission II of the Medical Faculty Mannheim, Heidelberg University, Heidelberg, Germany (reference number: 2011-292N-MA and 2023-851 respectively)[16].

Data preprocessing and outcome determination

The first measurement up to 24 hours after admission of 57 numerical features representing routine laboratory values, vital signs and interventions as well as clinical scores were included as potential predictors in the analysis (**Table S3**). These also included the criteria of the systemic inflammatory response syndrome (SIRS), which are derived from leucocyte count, heart rate, temperature and respiratory rate[17]. The SIRS-criteria were represented by descriptors which summarize SIRS over the 24 hours after admission as previously described[16]: in each patient, an algorithm determined the number of SIRS criteria met in each minute (λ). From this, the mean of 1440 minute λs was calculated for determination of 'SIRS average λ', the difference between λ of the last and the first minute of the 24 hours for 'SIRS Δ' and the number of changes in λ during the 24 hour period as 'SIRS C'. Multiple-imputation was applied to impute missing feature values. MCMC was used as the base model in the imputation, and 100 different imputed data tables were outputted and then averaged for the subsequent process. The imputation was performed using the internal functions of the software SAS V 9.4. Sepsis incidence was determined by retrospective review of the clinical data by medical experts. For the septic patients, the continuous coutcomes were determined as the last urea, creatinine and lactate measurements up to 24 hours before the sepsis onset time point. To determine the continuous outcome values of the non-septic polytrauma patients, we first created risk sets[18] at each time point a sepsis case was diagnosed during follow-up time in the ICU. The risk set contained the case and all non-cases with the same or longer duration of treatment in the ICU with respect to this time point. For each patient remaining sepsis-free until ICU discharge we took the meadian of all risks sets in which the individual patient had been included. If the median was between 2 risk sets, the earlier risk set, i.e. the one corresponding to the case with shorter time between admission and sepsis onset was chosen. The values that represented the last urea, creatinine and lactate measurements up to 24 hours before this time point were analyzed as continuous outcome parameters.

Before the machine learning stage, we performed a z-standardization on the feature matrix and the continuous outcomes.

Case study 2: Prediction of schizophrenia

Motivation

Schizophrenia, a severe mental illness, impacts approximately 24 million people worldwide, contributing to a massive social and economic burden[19]. Neuroimaging studies have shown that schizophrenia is associated with an accelerated aging of the brain. These studies are based on the concept of brain age — inferred from brain-structural or other data in contrast to chronological age — to quantify a potential acceleration of aging. This approach has uncovered evidence of age-dependent changes in brain structure [20], epigenetic data [21], and gene expression [22]. To further explore age-dependent effects in schizophrenia, we explore the joint prediction of age (regression task) and diagnosis (classification task) as an MTLComb case study. We assembled two cohorts comprising brain

expressions from individuals diagnosed with schizophrenia and healthy controls, designated as the discovery and validation cohorts. On the discovery cohort, an MTLComb model was employed to identify age-dependent genes that simultaneously serve as predictive markers for schizophrenia diagnosis. Subsequently, we assessed the reproducibility of these markers using a validation cohort and explored the associated pathways for biological interpretation.

Data cohorts

Four independent cortical expression cohorts were included in this study. The discovery dataset used for algorithm training was from the HBCC (Human Brain Collection Core) (n=422) cohort comprising genome-wide gene expression data quantified by microarray (dbGaP ID: phs000979.v3.p2). All patients included in the cohort met the Diagnostic and Statistical Manual of Mental Disorders, Fourth Edition (DSM-IV) criteria for a lifetime diagnosis of Axis I psychiatric disorders, such as schizophrenia or schizoaffective disorder, bipolar disorder, and major depression. RNA samples were obtained from the National Institute of Mental Health (NIMH) Human Brain Collection Core (HBCC). A detailed description of this dataset can be found in the original publication[23].

The validation dataset, comprising 194 subjects, integrates three cohorts: GSE53987[24], GSE21138[25], and GSE35977[26]. The diagnosis for each subject was made according to DSM-IV by experienced research clinicians. These data cohorts are publicly available for download from the Gene Expression Omnibus (GEO) repository. Detailed descriptions of these cohorts can be found on the GEO platform and in their respective original studies[24,26,27].

Data Preprocessing

The **discovery dataset** was normalised and quality controlled using the R package lumi 2.48.0[28]. First, we extracted Illumina BeadArray data from IDAT for HumanHT-12 v4 Gene Expression BeadChip based on the binary manifest file using the function LumiR.idat(). Then we selected samples from the DLPFC region, individuals aged 18-65 years, of either African American (AA) or Caucasian (CAUC) ethnicity. Here, a different procedure was applied to diagnosis-prediction and age-prediction tasks. For the diagnosis-prediction task, the data from all healthy controls and schizophrenia patients were used for the subsequent analysis, using age, age^2, race, RIN, sex, pH, PMI, 5 principle components and 5 surogate variables as covariates. For the age-prediction task, only healthy controls were investigated and ethnicity, RIN, sex, pH, PMI, 5 principle components and 5 surogate variable used as covariates. The following steps are applied individually for each task.

Next, we corrected for background noise using the lumiExpresso() function with quantile normalisation and log2 transformation parameters. We retained robustly expressed probes as those with a detection

p-value <0.01 in at least half of the individuals. We then excluded features with unknown expression (unavailable) and eliminated duplicate features. In order to eliminate duplicate features, we ordered them in descending order based on their median expression values across samples, selecting the highest value and discarding the others. Subsequently, the Ensembl IDs associated with the features were converted to gene symbols using the ensembl-based annotation R package (lumiHumanAll.db v1.22.0). Outlier samples deviating more than 4 standard deviations from the first or second PC were then removed. Prior to Surrogate Variable Analysis (SVA), missing values for the covariates pH and PMI were imputed using the mean of the available data. SVA was performed with control for the effects of the covariates. Then, PCs were calculated and the data adjusted for the top 5 PCs, the top 5 surrogate variables and the covariates. Finally, the resulting expression genes were z-standardized. The cohort comprised data for 201 healthy controls and 158 patients with schizophrenia that was used for the diagnosis-prediction task, and for 283 healthy controls for the age-prediction task. The demographics of these subjects are shown in **Table S1**. In the end, 9663 genes are included in the discovery cohort for in-cohort prediction test.

The **validation dataset** consists of three cohorts: GSE53987, GSE21138, and GSE35977. Initially, raw data were extracted using the ReadAffy() function from the R package affy[29]. This was followed by normalization via the Robust Multi-array Average (RMA) method[30]. Subsequently, probe IDs were converted into gene symbols for each cohort, tailored to the specific type of microarray chip used. For cohorts GSE53987 and GSE21138, the hgu133plus2.db database was employed, corresponding to data acquired on the Affymetrix GeneChip Human Genome U133 Plus 2.0 Array. For the GSE35977 cohort, which utilized the Affymetrix Human Gene 1.0 ST Array, the hugene10sttranscriptcluster.db database was used. Data alignment across the three cohorts involved mapping gene names and averaging values from multiple probes associated with the same gene. Subjects younger than 18 or older than 65 were excluded from the analysis. Distinct methodologies were then applied based on the prediction task: diagnosis-prediction task utilized data from both healthy controls and schizophrenia patients, incorporating covariates such as age, age squared, sex, pH, post-mortem interval (PMI), cohort index, five principal components, and five surrogate variables. Conversely, the age-prediction task was confined to healthy controls using the covariates: sex, pH, post-mortem interval (PMI), cohort index, five principal components, and five surrogate variables. The following steps are applied individually for each prediction task.

Outliers within each cohort were removed, defined as those exceeding four standard deviations from the mean along the first two principal components. Then, the cohorts were merged for surrogate variable analysis (SVA) using five surrogate variables. The top five principal components (PCs) were calculated for correction purposes. Then a linear regression analysis was utilized to adjust for potential

confounders, incorporating all covariates, five surrogate variables, and five PCs. Finally, the gene expression data were z-standardized. This process resulted in a dataset comprising 92 healthy controls and 93 schizophrenia patients for the diagnosis-prediction task, and 92 healthy controls for the age-prediction task. The demographics of these subjects are detailed in **Table S2**. Currently, 17,166 genes are under consideration. After matching with genes from the discovery cohort, 8,799 genes were retained in the validation cohort for cross-cohort prediction testing.

Parameter/Summary	Healthy controls		SCZ		Total	
	n	%	*n*	%	*n*	%
Total	201	56.0	158	44	359	100
Gender						
Female	58	28.9	55	34.8	113	31.5
Male	143	71.1	103	65.2	246	68.5
Race						
AA	108	53.7	65	41.1	173	48.2
CAUC	93	46.3	93	58.9	186	51.8
Age	*year*		*year*		*year*	
mean	43.0		45.9		44.3	
median	45.4		47.2		46.2	
min/max	18.0 / 65.0		18.0 / 63.2		18.0 / 65.0	
1st quantile	31.6		38.8		35.6	
3rd quantile	53.6		54.4		54.0	

Table S1: Demographics of the discovery dataset (HBCC)

Parameter/Summary	Healthy controls		SCZ		Total	
	n	%	*n*	%	*n*	%
Total	92	50	93	50	185	100
Gender						
Female	26	28	27	29.0	53	28.6
Male	66	72	66	71.0	132	71.4
Age	*year*		*year*		*year*	
mean	44.1		42.5		43.3	
median	45		45		45	
min/max	21.0 / 65.0		19.0 / 65.0		19.0 / 65.0	
1st quantile	37.8		35.0		36.0	
3rd quantile	50.0		50.0		50.0	

Table S2: Demongraphics of the validation dataset (GEO cohorts)

	Cohort 1 (N=242)			Cohort 2 (N=159)		
	Sepsis (N=78)	No sepsis (N=164)		Sepsis (N=56)	No sepsis (N=103)	
	Mean (SD) Median (IQR) n (%)	Mean (SD) Median (IQR) n (%)	p-value	Mean (SD) Median (IQR) n (%)	Mean (SD) Median (IQR) n (%)	p-value
Age [yrs]	50.8 (20.82)	47.0 (19.26)	0.1777	52.6 (18.88)	52.1 (20.96)	0.8912
Male	64 (82.1%)	120 (73.2%)	0.1304	47 (83.9%)	67 (65.0%)	0.0116
SAPSII*	29 (25–37)	25 (19–31)	<.0001#	32 (25–38)	29.5 (23–36)	0.0354#
ISS	41 (34–41)	34 (29–41)	<.0001#	29 (24–36)	26 (22–34)	0.0266#
AIS Head			0.5815~			0.5361~
0	33 (42.3%)	74 (45.1%)		14 (25.0%)	33 (32.0%)	
1	0 (0%)	1 (0.61%)		0 (0%)	1 (0.97%)	
2	3 (3.85%)	9 (5.49%)		3 (5.36%)	1 (0.97%)	
3	7 (8.97%)	24 (14.6%)		5 (8.93%)	7 (6.80%)	
4	34 (43.6%)	54 (32.9%)		31 (55.4%)	54 (52.4%)	
5	1 (1.28%)	2 (1.22%)		3 (5.36%)	7 (6.80%)	
AIS Face			0.9898~			0.5292~
0	40 (51.3%)	87 (53.0%)		30 (53.6%)	66 (64.1%)	
1	1 (1.28%)	2 (1.22%)		6 (10.7%)	9 (8.74%)	
2	10 (12.8%)	17 (10.4%)		19 (33.9%)	27 (26.2%)	
3	23 (29.5%)	48 (29.3%)		1 (1.79%)	1 (0.97%)	
4	4 (5.13%)	9 (5.49%)		0 (0%)	0 (0%)	
5	0 (0%)	1 (0.61%)		0 (0%)	0 (0%)	
AIS Thorax			0.0113~			0.1191~
0	11 (14.1%)	21 (12.8%)		11 (19.6%)	30 (29.1%)	
1	0 (0%)	1 (0.61%)		2 (3.57%)	6 (5.83%)	
2	1 (1.28%)	7 (4.27%)		5 (8.93%)	6 (5.83%)	
3	17 (21.8%)	66 (40.2%)		16 (28.6%)	38 (36.9%)	
4	49 (62.8%)	69 (42.1%)		20 (35.7%)	23 (22.3%)	
5	0 (0%)	0 (0%)		2 (3.57%)	0 (0%)	
AIS Abdomen			0.1300			0.1828~
0	37 (47.4%)	91 (55.5%)		28 (50.0%)	69 (67.0%)	
1	0 (0%)	0 (0%)		1 (1.79%)	0 (0%)	
2	3 (3.85%)	13 (7.93%)		14 (25.0%)	18 (17.5%)	
3	13 (16.7%)	30 (18.3%)		6 (10.7%)	7 (6.80%)	
4	23 (29.5%)	29 (17.7%)		7 (12.5%)	9 (8.74%)	
5	2 (2.56%)	1 (0.61%)				
AIS Extremities			0.8554~			0.7922~
0	14 (17.9%)	38 (23.2%)		16 (28.6%)	27 (26.2%)	
1	0 (0%)	1 (0.61%)		1 (1.79%)	3 (2.91%)	
2	4 (5.13%)	10 (6.10%)		15 (26.8%)	35 (34.0%)	
3	32 (41.0%)	63 (38.4%)		12 (21.4%)	23 (22.3%)	

	Cohort 1 (N=242)			Cohort 2 (N=159)		
	Sepsis (N=78)	No sepsis (N=164)		Sepsis (N=56)	No sepsis (N=103)	
	Mean (SD) Median (IQR) n (%)	Mean (SD) Median (IQR) n (%)	p-value	Mean (SD) Median (IQR) n (%)	Mean (SD) Median (IQR) n (%)	p-value
4	28 (35.9%)	52 (31.7%)		12 (21.4%)	14 (13.6%)	
5					1 (0.97%)	
AIS Soft tissue			0.1225			0.8869~
0	5 (6.41%)	15 (9.15%)		0 (0%)	1 (0.97%)	
1	0 (0%)	9 (5.49%)		20 (35.7%)	41 (39.8%)	
2	45 (57.7%)	98 (59.8%)		35 (62.5%)	59 (57.3%)	
3	25 (32.1%)	35 (21.3%)		1 (1.79%)	2 (1.94%)	
4	3 (3.85%)	7 (4.27%)		0 (0%)	0 (0%)	
Diabetes	10 (12.8%)	6 (3.66%)	0.0073	7 (12.5%)	13 (12.6%)	0.9824
Respiratory diseases	4 (5.13%)	6 (3.66%)	0.7310~	3 (5.36%)	1 (0.97%)	0.1257~
Alcoholism	14 (17.9%)	17 (10.4%)	0.0990	12 (21.4%)	10 (9.71%)	0.0409
Cardiovascular diseases	15 (19.2%)	17 (10.4%)	0.0571	17 (30.4%)	9 (8.74%)	0.0004
Body temperature [°C]	35.7 (1.45)	36.2 (1.25)	0.0094	36.1 (1.05)	36.0 (1.35)	0.5294
pH*	7.338 (0.08)	7.351 (0.07)	0.2035	7.334 (0.10)	7.369 (0.06)	0.0169
Lactate [mmol/L]*	2.34 (1.74)	1.90 (1.13)	0.0403	2.16 (1.51)	1.91 (1.19)	0.2861
Base excess [mmol/L]*	-1.5 (3.43)	-1.0 (2.77)	0.3207	-2.1 (3.09)	-1.1 (2.47)	0.0427
pO2 [mmHg]*	177.5 (109.18)	160.2 (89.43)	0.2274	171.9 (105.44)	151.8 (82.11)	0.2226
pCO2 [mmHg]*	45.2 (10.31)	44.6 (8.32)	0.6897	47.6 (21.21)	42.3 (7.28)	0.0738
Calcium [mmol/L]*	1.1 (0.11)	1.1 (0.08)	0.2192	1.2 (0.08)	1.2 (0.09)	0.3549
Potassium [mmol/L]*	3.9 (0.59)	3.9 (0.46)	0.7909	4.0 (0.54)	4.0 (0.44)	0.4369
FiO2 [%]	55.3 (22.59)	47.1 (23.97)	0.0104	47.5 (19.35)	38.4 (17.43)	0.0040
Mechanical Ventilation	67 (85.9%)	109 (66.5%)	0.0015	48 (85.7%)	70 (68.0%)	0.0145
WBC [10E9/L]*	9.08 (3.96)	10.56 (4.76)	0.0121	11.98 (4.98)	10.69 (3.71)	0.0954
Hb [g/dL]	9.65 (2.39)	10.93 (2.20)	0.0001	10.71 (2.14)	11.02 (2.08)	0.3908
Hematocrit [%]*	27.72 (6.63)	31.06 (5.41)	0.0002	28.54 (6.53)	30.85 (5.72)	0.0284
Erythrocytes [10E12/L]*	3.2 (0.75)	3.6 (0.62)	0.0002	3.3 (0.72)	3.5 (0.65)	0.0171
Thrombocytes [10E9/L]*	120.7 (47.24)	160.6 (63.05)	<.0001	158.3 (63.22)	168.6 (52.27)	0.3027

	Cohort 1 (N=242)			Cohort 2 (N=159)		
	Sepsis (N=78)	No sepsis (N=164)		Sepsis (N=56)	No sepsis (N=103)	
	Mean (SD) Median (IQR) n (%)	Mean (SD) Median (IQR) n (%)	p-value	Mean (SD) Median (IQR) n (%)	Mean (SD) Median (IQR) n (%)	p-value
INR*	1.2 (0.29)	1.1 (0.19)	0.0290	1.2 (0.40)	1.1 (0.17)	0.1544
pTT [sec]*	30.2 (8.20)	26.8 (4.00)	0.0006	26.7 (4.60)	26.0 (4.32)	0.3539
Mean arterial pressure (MAP) [mmHg]	88.2 (18.26)	89.9 (16.41)	0.4928	84.4 (17.36)	86.6 (18.75)	0.4538
Systolic blood pressure [mmHg]	125.4 (27.36)	130.1 (22.23)	0.1858	122.3 (27.38)	128.2 (32.44)	0.2217
Diastolic blood pressure [mmHg]	68.72 (14.54)	70.95 (13.88)	0.2604	66.55 (13.97)	68.76 (16.08)	0.3699
Heart rate [1/min]	91.6 (21.21)	88.9 (18.87)	0.3275	90.8 (21.79)	86.9 (23.50)	0.2862
Catecholamines	34 (43.6%)	33 (20.1%)	0.0001	44 (78.6%)	45 (43.7%)	<.0001
Shock index	0.78 (0.28)	0.70 (0.20)	0.0489	0.78 (0.27)	0.71 (0.25)	0.1266
Volume balance [L]*	4.25 (2.70)	3.64 (2.73)	0.1184	4.09 (3.30)	3.03 (2.83)	0.0444
Bilirubin [mg/dL]	0.76 (0.45)	0.65 (0.38)	0.0851	0.76 (0.50)	0.72 (0.45)	0.6743
Glucose [mg/dL]*	146.0 (45.10)	136.1 (36.25)	0.0930	146.9 (40.84)	140.8 (41.99)	0.3738
Creatinine [mg/dL]*	0.96 (0.35)	0.90 (0.26)	0.1666	1.06 (0.42)	0.97 (0.34)	0.1604
Urea [mg/dL]*	33.5 (21.17)	28.8 (12.15)	0.0735	33.5 (13.64)	33.0 (14.97)	0.8335
Urea/Creatinine*	34.8 (12.84)	32.8 (12.19)	0.2769	32.8 (10.51)	34.6 (12.17)	0.3348
CRP determined	24 (30.8%)	48 (29.3%)	0.8113	54 (96.4%)	91 (88.3%)	0.1406~
PCT determined	21 (26.9%)	15 (9.15%)	0.0003	12 (21.4%)	7 (6.80%)	0.0066
GCS	12 (4–15)	13 (8–15)	0.0109#	11 (3–14)	14 (7–15)	0.0127#
EK	43 (55.1%)	52 (31.7%)	0.0005	22 (39.3%)	22 (21.4%)	0.0158
SOFA Total*	9 (7–11)	6 (4–9)	<.0001#	9 (6–10)	6 (4–9)	0.0002#
SOFA Respiratory*			0.0054~			0.7455~
0	0 (0%)	6 (3.66%)		1 (1.79%)	3 (2.91%)	
1	9 (11.5%)	24 (14.6%)		6 (10.7%)	8 (7.77%)	
2	14 (17.9%)	49 (29.9%)		18 (32.1%)	43 (41.7%)	
3	33 (42.3%)	65 (39.6%)		27 (48.2%)	41 (39.8%)	
4	22 (28.2%)	18 (11.0%)		4 (7.14%)	8 (7.77%)	
SOFA Cardiovascular			0.0004			0.0026
0	14 (17.9%)	56 (34.1%)		7 (12.5%)	21 (20.4%)	
1	30 (38.5%)	75 (45.7%)		8 (14.3%)	38 (36.9%)	

	Cohort 1 (N=242)			Cohort 2 (N=159)		
	Sepsis (N=78)	No sepsis (N=164)		Sepsis (N=56)	No sepsis (N=103)	
	Mean (SD) Median (IQR) n (%)	Mean (SD) Median (IQR) n (%)	p-value	Mean (SD) Median (IQR) n (%)	Mean (SD) Median (IQR) n (%)	p-value
3	8 (10.3%)	13 (7.93%)		13 (23.2%)	16 (15.5%)	
4	26 (33.3%)	20 (12.2%)		28 (50.0%)	28 (27.2%)	
SOFA Coagulation*			<.0001~			0.3708~
0	13 (16.7%)	72 (43.9%)		28 (50.0%)	60 (58.3%)	
1	23 (29.5%)	49 (29.9%)		18 (32.1%)	28 (27.2%)	
2	38 (48.7%)	37 (22.6%)		7 (12.5%)	12 (11.7%)	
3	4 (5.13%)	5 (3.05%)		3 (5.36%)	1 (0.97%)	
SOFA Renal*			0.5654~			0.5502~
0	63 (80.8%)	141 (86.0%)		42 (75.0%)	79 (76.7%)	
1	14 (17.9%)	21 (12.8%)		12 (21.4%)	21 (20.4%)	
2	1 (1.28%)	1 (0.61%)		2 (3.57%)	1 (0.97%)	
SOFA Hepatic			0.0107~			0.5226~
0	56 (71.8%)	139 (84.8%)		47 (83.9%)	93 (90.3%)	
1	13 (16.7%)	21 (12.8%)		7 (12.5%)	8 (7.77%)	
2	7 (8.97%)	4 (2.44%)		2 (3.57%)	2 (1.94%)	
3	2 (2.56%)	0 (0%)		0 (0%)	0 (0%)	
SOFA Neuro			0.0720			0.0895
0	24 (30.8%)	73 (44.5%)		12 (21.4%)	43 (41.7%)	
1	12 (15.4%)	20 (12.2%)		10 (17.9%)	17 (16.5%)	
2	6 (7.69%)	22 (13.4%)		8 (14.3%)	9 (8.74%)	
3	13 (16.7%)	20 (12.2%)		7 (12.5%)	13 (12.6%)	
4	23 (29.5%)	29 (17.7%)		19 (33.9%)	21 (20.4%)	
SIRS average λ	1.70 (0.67)	1.28 (0.73)	<.0001	1.74 (0.65)	1.36 (0.71)	0.0012
SIRS count C	14.5 (10.44)	17.7 (11.08)	0.0303	13.9 (6.57)	15.3 (9.20)	0.2763
SIRS Δ	0.5 (1.36)	0.2 (1.36)	0.1074	0.9 (1.16)	0.8 (1.21)	0.5596

The statistics of outcomes measured at onset time point

	Cohort 1 (N=242)			Cohort 2 (N=159)		
	Sepsis (N=78)	No sepsis (N=164)		Sepsis (N=56)	No sepsis (N=103)	
	Mean (SD)	Mean (SD)	p-value	Mean (SD)	Mean (SD)	p-value
Creatinine [mg/dL]*	1.04 (0.49)	0.81 (0.37)	0.00027	1.07 (0.53)	0.92 (0.49)	0.088
Urea [mg/dL]*	59.9 (35.5)	33 (17.6)	<.0001	57.4 (35.3)	41.2 (25)	0.0032
Lactate [mmol/L]*	1.2 (0.76)	0.83 (0.33)	<.0001	1.1 (0.56)	0.88 (0.38)	0.0069

Table S3: Statistical Summary of Features and Outcomes in Sepsis Analysis. This table presents a statistical summary of the features and outcomes used in the analysis of sepsis. The p-values referred to the significance of differences between the sepsis and non-sepsis groups. Features that contain missing data are marked with an asterisk (*). The method of p-value calculation varies depending on the data type: a t-test is used for continuous features, while a Chi-squared (χ^2) test is used for categorical features. P-values calculated using the Mann-Whitney-Wilcoxon test are denoted by a hash (#), and those calculated using Fisher's exact test are indicated by a tilde (~).

Pathway ID	Pathway name	FDR value
GO:0005244	voltage-gated monoatomic ion channel activity	0.003115691
GO:0022832	voltage-gated channel activity	0.003115691
GO:0022832	gated channel activity	0.007911141
GO:0022839	monoatomic ion gated channel activity	0.007911141
GO:0007268	chemical synaptic transmission	0.025878397
GO:0098916	anterograde trans-synaptic signaling	0.025878397
GO:0099537	trans-synaptic signaling	0.025878397
GO:0009887	animal organ morphogenesis	0.025878397
GO:0030001	metal ion transport	0.025878397
GO:0006469	negative regulation of protein kinase activity	0.025878397
GO:0007423	sensory organ development	0.032789873
GO:0099536	synaptic signaling	0.033531818
GO:0033673	negative regulation of kinase activity	0.046850028

Table S4: The enriched pathways using top selected genes of MTLComb model from Schizophrenia analysis.

References

1. Liu J, Ji S, Ye J. Multi-task feature learning via efficient l2, 1-norm minimization. 2009:339-348.
2. Evgeniou T, Pontil M. Regularized multi-task learning. ACM; 2004:109-117.
3. Cao H, Schwarz E. An Tutorial for Regularized Multi-task Learning using the package RMTL. The Comprehensive R Archive Network.
4. Zou H, Hastie T. Regularization and variable selection via the elastic net. *Journal of the Royal Statistical Society: Series B (Statistical Methodology)*. 2005;67(2):301-320. doi:10.1111/j.1467-9868.2005.00503.x
5. Boyd S, Vandenberghe L. *Convex optimization*. Cambridge university press; 2004.
6. Nesterov Y. Gradient methods for minimizing composite functions. *Mathematical Programming*. 2012;140(1):125-161. doi:10.1007/s10107-012-0629-5
7. Beck A, Teboulle M. A fast iterative shrinkage-thresholding algorithm for linear inverse problems. *SIAM journal on imaging sciences*. 2009;2(1):183-202.
8. Liu J, Jieping Y. Efficient L1/Lq Norm Regularization.
9. Blanco J, Muriel-Bombín A, Sagredo V, et al. Incidence, organ dysfunction and mortality in severe sepsis: a Spanish multicentre study. *Critical care*. 2008;12:1-14.
10. Fleischmann C, Scherag A, Adhikari NK, et al. Assessment of global incidence and mortality of hospital-treated sepsis. Current estimates and limitations. *American journal of respiratory and critical care medicine*. 2016;193(3):259-272.
11. Singer M, Deutschman CS, Seymour CW, et al. The third international consensus definitions for sepsis and septic shock (Sepsis-3). *Jama*. 2016;315(8):801-810.

12. Kong G, Lin K, Hu Y. Using machine learning methods to predict in-hospital mortality of sepsis patients in the ICU. *BMC medical informatics and decision making*. 2020;20:1-10.
13. Kim HI, Park S. Sepsis: early recognition and optimized treatment. *Tuberculosis and respiratory diseases*. 2019;82(1):6-14.
14. Farzanegan B, Zangi M. Predictor factors for sepsis diagnosis, length of ICU stay and mortality in ICU. *Journal of Cellular & Molecular Anesthesia*. 2017;2(2):55-62.
15. Alsinglawi B, Alnajjar F, Mubin O, Novoa M, Karajeh O, Darwish O. Benchmarking predictive models in electronic health records: Sepsis length of stay prediction. Springer; 2020:258-267.
16. Lindner HA, Balaban U, Sturm T, Weiss C, Thiel M, Schneider-Lindner V. An Algorithm for Systemic Inflammatory Response Syndrome Criteria-Based Prediction of Sepsis in a Polytrauma Cohort. *Crit Care Med*. Dec 2016;44(12):2199-2207. doi:10.1097/CCM.0000000000001955
17. Bone RC, Balk RA, Cerra FB, et al. Definitions for sepsis and organ failure and guidelines for the use of innovative therapies in sepsis. The ACCP/SCCM Consensus Conference Committee. American College of Chest Physicians/Society of Critical Care Medicine. *Chest*. Jun 1992;101(6):1644-55. doi:10.1378/chest.101.6.1644
18. Langholz B, Goldstein L. Risk set sampling in epidemiologic cohort studies. *Statistical Science*. 1996:35-53.
19. Chong HY, Teoh SL, Wu DB-C, Kotirum S, Chiou C-F, Chaiyakunapruk N. Global economic burden of schizophrenia: a systematic review. *Neuropsychiatric disease and treatment*. 2016:357-373.
20. Kaufmann T, van der Meer D, Doan NT, et al. Common brain disorders are associated with heritable patterns of apparent aging of the brain. *Nature neuroscience*. 2019;22(10):1617-1623.
21. Saarinen A, Marttila S, Mishra PP, et al. Polygenic risk for schizophrenia, social dispositions, and pace of epigenetic aging: Results from the Young Finns Study. *Aging Cell*. 2023:e14052.
22. Lin C-W, Chang L-C, Ma T, et al. Older molecular brain age in severe mental illness. *Molecular psychiatry*. 2021;26(7):3646-3656.
23. Fromer M, Roussos P, Sieberts SK, et al. Gene expression elucidates functional impact of polygenic risk for schizophrenia. Article. *Nat Neurosci*. 11//print 2016;19(11):1442-1453. doi:10.1038/nn.4399 http://www.nature.com/neuro/journal/v19/n11/abs/nn.4399.html#supplementary-information
24. Lanz TA, Reinhart V, Sheehan MJ, et al. Postmortem transcriptional profiling reveals widespread increase in inflammation in schizophrenia: a comparison of prefrontal cortex, striatum, and hippocampus among matched tetrads of controls with subjects diagnosed with schizophrenia, bipolar or major depressive disorder. *Transl Psychiatry*. May 23 2019;9(1):151. doi:10.1038/s41398-019-0492-8
25. Narayan S, Tang B, Head SR, et al. Molecular profiles of schizophrenia in the CNS at different stages of illness. *Brain Res*. Nov 06 2008;1239:235-48. doi:10.1016/j.brainres.2008.08.023
26. Chen C, Cheng L, Grennan K, et al. Two gene co-expression modules differentiate psychotics and controls. *Mol Psychiatry*. Dec 2013;18(12):1308-14. doi:10.1038/mp.2012.146
27. Tang B, Capitao C, Dean B, Thomas EA. Differential age- and disease-related effects on the expression of genes related to the arachidonic acid signaling pathway in schizophrenia. *Psychiatry Res*. Apr 30 2012;196(2-3):201-6. doi:10.1016/j.psychres.2011.09.026
28. Du P, Kibbe WA, Lin SM. lumi: a pipeline for processing Illumina microarray. *Bioinformatics*. 2008;24(13):1547-1548.
29. Gautier L, Cope L, Bolstad BM, Irizarry RA. affy--analysis of Affymetrix GeneChip data at the probe level. *Bioinformatics*. Feb 12 2004;20(3):307-15. doi:10.1093/bioinformatics/btg405
30. Bolstad BM, Irizarry RA, Astrand M, Speed TP. A comparison of normalization methods for high density oligonucleotide array data based on variance and bias. *Bioinformatics*. Jan 22 2003;19(2):185-93. doi:10.1093/bioinformatics/19.2.185
